# Supplementary material for: Is Dairy Effluent an Alternative for Maize Crop Fertigation in Semiarid Regions? An Approach to Agronomic and Environmental Effects
Source: Animals (Basel). 2022 Aug 10;12(16):2025. doi: 10.3390/ani12162025 (PMC9404449; doi:10.3390/ani12162025)
Supplement: Supplementary file 1 [file animals-12-02025-s001.zip › animals-1832826-supplementary.pdf]

Supplementary Materials

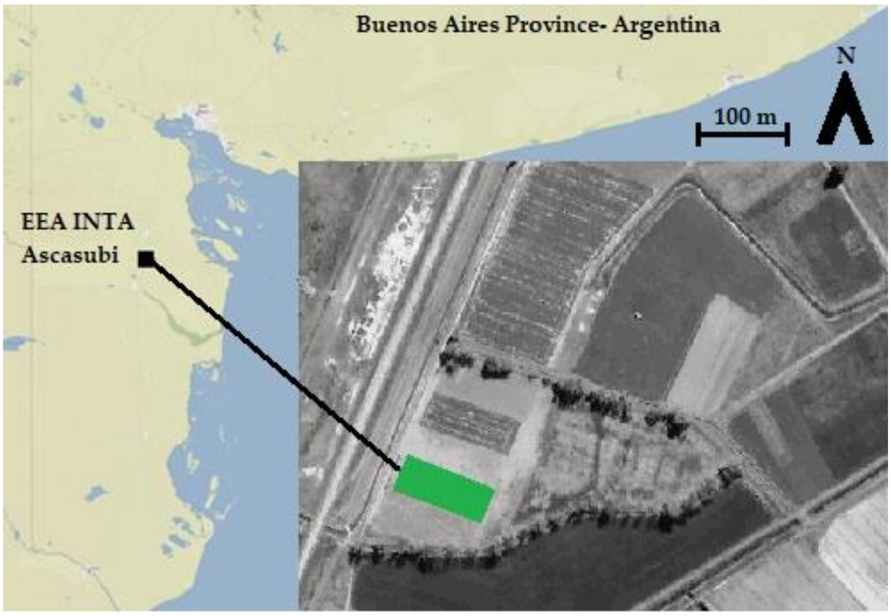

Figure S1. Map of the study area, showing the location of the experimental site on a high-resolution LANDSAT image (Hilario Ascasubi, Buenos Aires province, Argentina 2020).

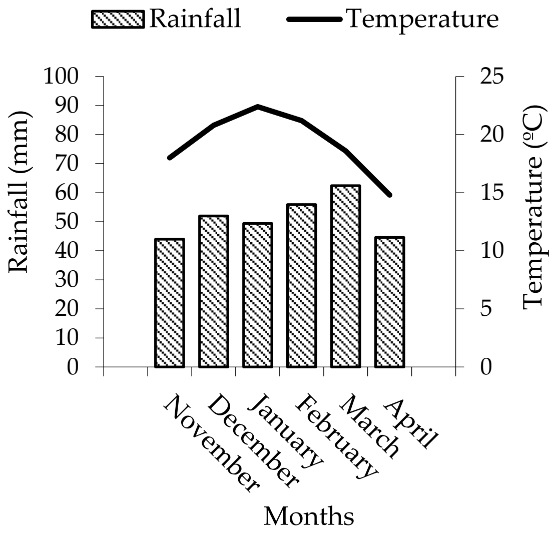

(a)

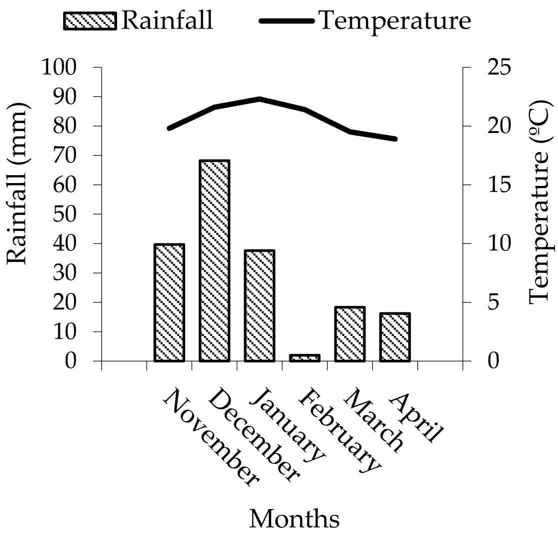

(b)

Figure S2. Mean monthly temperature and rainfall at the experimental site: **(a)** historical (November 1966 - April 2021) and **(b)** during the experimental period (November 2020- April 2021).

Table S1. Physicochemical soil parameters in two sampling moments (0-60 cm), before the liquid fertilization (V2 stage, "start") and after the conclusion of the maize cycle (harvest, "end").

**Soil depth** (cm)	**Treatment**	**Sampling moment**	**pH**	**EC** ($dS\ m^{-1}$)	**eP** ($mg\ kg^{-1}$)	**OM** (%)
0-10	C	Start	6.58 ± 0.15 a	1.85 ± 0.13 a	49.10 ± 11.29 a	2.99 ± 0.16 a
		End	7.01 ± 0.15 a	2.00 ± 0.13 a	28.70 ± 11.29 a	2.68 ± 0.16 a
	DE	Start	6.59 ± 0.10 a	1.50 ± 0.16 a	44.28 ± 5,55 a	2.69 ± 0.14 a
		End	6.49± 0.10 a	2.12 ± 0.16 a	37.45 ± 5,55 a	2.98 ± 0.14 a
	U	Start	6.80 ± 0.05 a	1.51 ± 0.19 a	31.13 ± 1.69 a	2.65 ± 0.27 a
		End	6.58 ± 0.05 a	1.96 ± 0.19 a	25.98 ± 1.69 a	2.96 ± 0.27 a
10-20	C	Start	7.07 ± 0.03 a	1.43 ± 0.08 a	10.20 ± 1.73 a	2.24 ± 0.12 a
		End	7.48 ± 0.03 b	1.41 ± 0.08 a	4.20 ± 1.73 a	1.87 ± 0.12 a
	DE	Start	7.17 ± 0.04 a	1.32 ± 0.06 a	7.05 ± 1.05 a	1.79 ± 0.04 a
		End	6.99 ± 0.04 a	1.41 ± 0.06 a	5.73 ± 1.05 a	1.93 ± 0.04 a
	U	Start	7.25 ± 0.06 b	1.27 ± 0.05 a	7.45 ± 0.55 a	1.97 ± 0.08 a
		End	6.88 ± 0.06 a	1.57 ± 0.05 b	5.58 ± 0.55 a	2.18 ± 0.08 a
20-60	C	Start	8.14 ± 0.14 a	1.51 ± 0.11 a	2.98 ± 0.26 a	1.87 ± 0.11 a
		End	8.24 ± 0.14 a	1.62 ± 0.11 a	3.45 ± 0.26 a	1.97 ± 0.11 a
	DE	Start	8.13 ± 0.16 a	1.45 ± 0.06 a	3.28 ± 0.19 a	1.47 ± 0.02 a
		End	8.32 ± 0.16 a	1.81 ± 0.06 b	2.58 ± 0.19 a	1.93 ± 0.02 b
	U	Start	8.10 ± 0.10 a	1.54 ± 0.07 a	3.50 ± 0.44 a	1.68 ± 0.07 a
		End	8.04 ± 0.10 a	1.73 ± 0.07 a	2.95 ± 0.44 a	1.95 ± 0.07 b

EC: electrical conductivity; eP: extractable phosphorus; OM: organic matter. Mean values ± standard error. Different letters indicate differences between sampling day (start and end) for each treatment ($p<0.05$).
